# *Plasmodium* metabolite HMBPP stimulates feeding of main mosquito vectors on blood and artificial toxic sources

**DOI:** 10.1038/s42003-021-02689-8

**Published:** 2021-10-07

**Authors:** Viktoria E. Stromsky, Melika Hajkazemian, Elizabeth Vaisbourd, Raimondas Mozūraitis, S. Noushin Emami

**Affiliations:** 1 Department of Molecular Biosciences, Wenner-Gren Institute, Stockholm University, Stockholm, Sweden; 2 Laboratory of Chemical and Behavioural Ecology, Institute of Ecology, Nature Research Centre, Vilnius, Lithuania; 3 Department of Zoology, Stockholm University, Stockholm, Sweden; 4 Molecular Attraction AB, Elektravägen 10, 126 30 Hägersten, Stockholm, Sweden; 5 Natural Resources Institute, FES, University of Greenwich, London, UK

**Keywords:** Behavioural methods, Natural products

## Abstract

Recent data show that parasites manipulate the physiology of mosquitoes and human hosts to increase the probability of transmission. Here, we investigate phagostimulant activity of *Plasmodium-*metabolite, (*E*)-4-hydroxy-3-methyl-but-2-enyl pyrophosphate (HMBPP), in the primary vectors of multiple human diseases, *Anopheles coluzzii*, *An. arabiensis*, *An. gambiae* s.s., *Aedes aegypti*, and *Culex pipiens/Culex torrentium* complex species. The addition of 10 µM HMBPP to blood meals significantly increased feeding in all the species investigated. Moreover, HMBPP also exhibited a phagostimulant property in plant-based-artificial-feeding-solution made of beetroot juice adjusted to neutral pH similar to that of blood. The addition of AlbuMAX^TM^ as a lipid/protein source significantly improved the feeding rate of *An. gambiae* s.l. females providing optimised plant-based-artificial-feeding-solution for delivery toxins to control vector populations. Among natural and synthetic toxins tested, only fipronil sulfone did not reduce feeding. Overall, the toxic-plant-based-artificial-feeding-solution showed potential as an effector in environmentally friendly vector-control strategies.

## Abstract (web summary)

Viktoria Stromsky and Melika Hajkazemian et al. examine the potential effect of HMPBB, a *Plasmodium* metabolite, on blood-feeding rates of major vector mosquitoes, like *Anopheles gambiae*. They report that HMBPP significantly increased blood feeding on all tested mosquito species, and suggest that it might be used to stimulate mosquitoes to gorge themselves on toxic baits, and ultimately die.

## Introduction

Half of the world’s population is at risk from mosquito-borne diseases^1^. Quality-assured vector control is identified by the World Health Organization (WHO) as one of the three main strategies to control malaria^2^. Malaria imposes a huge health burden on the world’s most vulnerable population, with children under the age of five the most vulnerable. At present, the best defence is pesticides, nets or repellents. While these measures are only partially effective and have additional drawbacks, the vectors are becoming resistant and altering their behaviour. These trends combined with the global epidemiological burden of vector-borne diseases necessitate a search for new vector-control approaches that reduce vector-to-host transmission capabilities^1,3^.

Long-lasting insecticide-treated nets (LLITNs) and indoor residual spraying (IRS) contribute significantly to preventing human contact with malaria vectors and to decreasing malaria cases^4,5^. However, mosquitoes show high physiological and ecological plasticity and quickly adapt to environmental changes^6–8^, which leads to increasing insecticide resistance and decreasing effectiveness of LLITNs and IRS^9,10^. Moreover, diurnal fluctuation in females host-seeking behaviour has been reported in several main malaria vectors. Some anopheline mosquitoes now target human hosts during waking hours, when bed nets are ineffective^11^. It has also been reported that some species changed their host-seeking behaviour from indoors to outdoors^12–16^. Our current vector-control tools mainly target *Anopheles* vectors that feed and rest indoors at night^5^.

Development of innovative control methods such as the sterile insect technique (SIT), biotechnological control agents (e.g. endosymbionts), insects carrying a dominant lethal gene (RIDL/gene drive) as well as acoustic larvicides, RNAi-based bioinsecticides, and semiochemicals, i.e. compounds mediating info-chemical interactions between organisms, exhibit increasing success in vector-control strategies combined with low risk of emerging resistance^4,17–20^. Vector control is a complex battlefield, and it cannot be achieved by a single method; hence, integrated vector management programmes are essential for targeting vectors at different life stages or/and at various adult mosquito behaviour^21^.

The attractive toxic sugar bait (ATSB) technique exploits the sugar feeding behaviour of insects, particularly mosquitoes. Male mosquitoes feed exclusively on sugars while females take a carbohydrate meal before or after blood feeding^22^. Mosquitoes locate sugar sources through visual and olfactory cues. The sugar engorgement is stimulated by tarsal contact with sugars. In the ATSB, the sugar source is blended with a toxin and plant volatiles which are usually used to lure mosquitoes towards baits^23^. However, field studies evaluating the effects of ATSB on non-target arthropods revealed that insects from six major orders consumed and were affected by the toxic sugar mix, including eco-economically important insects^23,24^. One solution for the ATSB’s drawback is to replace the sugar, which enhances the feeding of non-target arthropods, with more species-specific phagostimulants, which would decrease the harmful effect of attract and kill methods.

Most blood-sucking insects have one phagostimulant in common, adenosine triphosphate (ATP)^25,26^. ATP in physiological saline has been reported to stimulate gorging^27^ in mosquitoes^28–31^, tsetse flies^32^, tabanids^33^, simulids^26^, fleas^34^ and *Rhodnius*^35^. However, ATP degrades at room temperature and is relatively unstable in aqueous solutions^36^. Finding a replacement for ATP is of great importance. The accumulating data show that parasites manipulate host and vector physiology to increase the probability of transmission^37–40^, which could be exploiteded for innovative vector-control methods. The specific requirement is to develop a vector-control method that targets all hematophagous disease vectors and is independent of species identity, urban or rural environment, and the host preference indoor/outdoor biting. To develop a universal method for the delivery of control agents to vectors, one must first identify a behaviour, characterise the underlying mechanism, bring the vector in close contact with the agent, and then exploit it.

Recently, a compelling piece of evidence found that malaria parasite, *Plasmodium falciparum* Welch (Haemospororida: Plasmodiidae), releases a semiochemical, (*E*)-4-hydroxy-3-methyl-but-2-enyl pyrophosphate (HMBPP) into blood. Addition of HMBPP in blood and serum meals or even in physiological saline, is sufficient to stimulate 80–100% of the *An. gambiae* sensu lato (s.l.) to gorge^41^, double the number that would gorge on a blood or saline meal alone. HMBPP is an intermediate metabolite from 2-C-methyl-D-erythritol 4-phosphate (MEP) biosynthetic pathway providing building blocks for isoprenoids in some bacteria, plants and eukaryotic apicomplexa, including *Plasmodium* parasites^42^. Given the crucial importance of parasite manipulation for enhancing its transmission, here, we hypothesise that the *Plasmodium* metabolite, HMBPP, increases the mosquito feeding proportion. Accordingly, the direct phagostimulatory effect of HMBPP when included in a toxic-plant-based artificial-feeding solution could be used to fight against multiple mosquito vectors. To test this hypothesis, we first determined the phagostimulatory activity of HMBPP in blood meals among main mosquito vectors, *An. coluzzii*, *An. arabiensis*, *An. gambiae* s.s., *Ae. aegypti* and *Cx. pipiens/Cx. torrentium*. Then, we have optimised a plant-based artificial feeding solution for delivery of natural and synthetic toxins as effectors to kill mosquito vectors. The data showing universal phagostimulatory activity of HMBPP to main malaria vectors provides valuable information for the use of this phagostimulant in vector-control strategies.

## Results

### HMBPP stimulates the mosquito feeding behaviour in main mosquito vectors

**Fig. 1:**
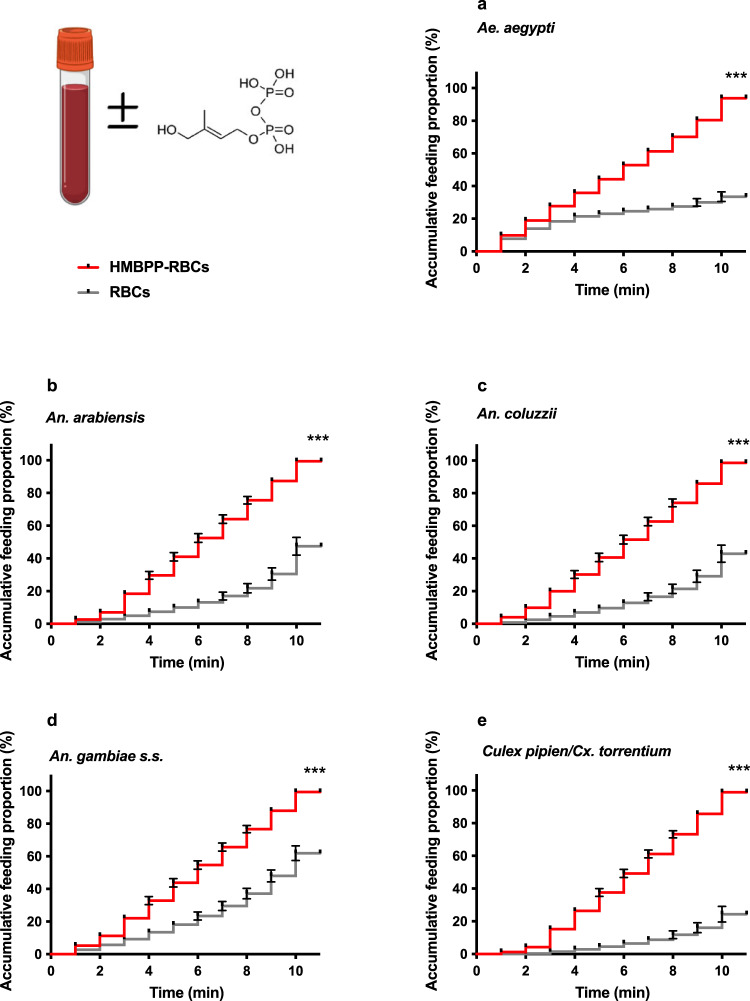
The direct effect of HMBPP on vectors accumulative feeding proportion. **a**–**e** Feeding proportions of mosquito vectors allowed to feed for 10 min on RBCs, or HMBPP-supplemented RBCs (each level of estimation generated from cox proportional hazard model ± SE; RBCs vs HMBPP-RBCs). Percentage of mosquitoes initiating feeding within 10 min on RBCs with and without HMBPP (RBCs vs HMBP-PRBCs) in all five vector species [*Ae. aegypti*: *χ*^2^_1_ = 183.6, *p* < 0.001; *Culex pipiens/Cx. torrentium*: *χ*^2^_1_ = 283.7, *p* < 0.001; *An. gambiae s.s*.: *χ*^2^_1_ = 212.4, *p* < 0.001; *An. arabiensis*: *χ*^2^_1_ = 210.2, *p* < 0.001; *An. coluzzii:* *χ*^2^_1_ = 138.1, *p* < 0.001]. Error bars, ±SE; asterisks denote significant differences (*n* = 120 per species, *p < 0.05, **p < 0.01, ***p < 0.001).

An increased propensity of female mosquitoes of all the tested species to land and feed on membrane feeders containing 10 µM HMBPP-supplemented red blood cells (HMBPP-RBCs), compared to control red blood cells (RBCs), was observed. This experiment revealed that HMBPP acted as a phagostimulant for all tested mosquitoes (beta estimation ± SE (b-glmer ± SE): *Ae. aegypti*: *χ*^2^_1_ = 99.60, *p* < 0.001; *Cx. pipiens/Cx. torrentium*: *χ*^2^_1_ = 124.02, *p* < 0.001; *An. gambiae* s.s.: *χ*^2^_1_ = 99.16, *p* < 0.001; *An. arabiensis*: *χ*^2^_1_ = 92.36, *p* < 0.001; *An. coluzzii:* *χ*^2^_1_ = 96.68, *p* < 0.001) as the proportion of fed mosquitoes doubled when offered HMBPP-RBCs rather than RBCs. To further decipher the phagostimulatory action of HMBPP, we provided the blood meals to mosquitoes and examined the percentage of females that landed and initiated probing and feeding within 10 min (Fig. 1a–e). Over 90% of the mosquitoes fed on HMBPP-supplemented blood compared to 20–60% from human blood alone (Fig. 1a–e). Hence, HMBPP acted as a phagostimulant to all five vector species with a small variation between species in the time for initiating feeding. The pyrophosphate group is necessary for the phagostimulatory effect, as (2*E*)-2-methylbut-2-ene-1,4-diol, part of the HMBPP molecule without pyrophosphate moiety, displayed no phagostimulatory effect (*χ*^2^_1_ = 0.25, *p* = 0.62; Supplementary Fig. 3). In addition, we found a significant difference in feeding proportion between species when they offered blood without HMBPP (*χ*^2^_4_ = 12.45, *p* < 0.001), which disappeared when the blood is supplemented with HMBPP (*χ*^2^_1_ = 0.25, *p* = 0.62). This experiment also demonstrated that there was significant difference among species for initiating time of the feed in control groups (without HMBPP). *An. gambiae* s.s. initiated the blood feeding with fastest record of <1 min, followed by the *Ae. aegypti*, *An. coluzzii*, *An. arabiensis* and *Culex* (Supplementary Fig. 4; *An. gambiae s.s*. vs *Ae. aegypti*: *z* = 3.67, *p* = 0.002; *Ae. aegypti* vs *An. arabiensis:* *z* = 3.60, *p* = 0.001; *An. arabiensis* vs *An. coluzzii:* *z* = 0.16, *p* = 0.60; *An. coluzzii* vs *Cx. pipiens/Cx. torrentium:* *z* = −4.89, *p* < 0.001). However, variation in the time of detection and initiation of the feeder among species vanished when the blood is supplemented with HMBPP (*χ*^2^_4_ = 7.20, *p* = 0.12).

### HMBPP stimulates the mosquito feeding behaviour on plant-based solution with optimal pH

**Fig. 2:**
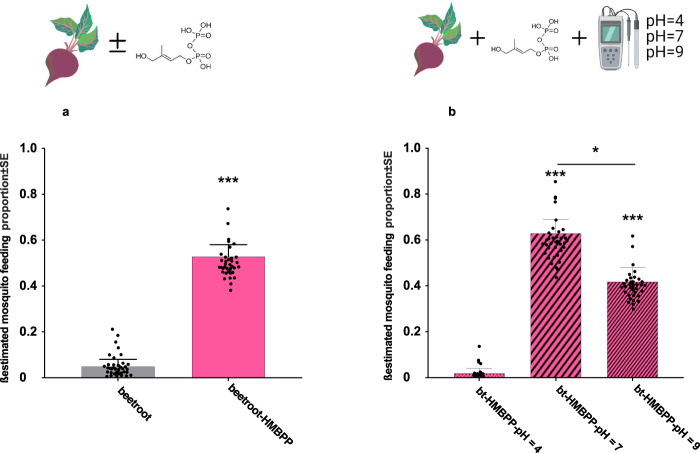
Mosquito feeding on a plant-based solution based on beetroot juice containing phagostimulant, HMBPP adjusted to various pH. **a** Percentage of *An. gambiae* s.l. feeding within ten minutes on beetroot juice with and without HMBPP (beetroot vs beetroot-HMBPP *χ*^2^_1_ = 12.05, *p* < 0.001). **b** Percentage of *An. gambiae* s.l. mosquitoes feeding within ten minutes on plant-based solution containing HMBPP (bt-HMBPP) with three different acidity levels (bt-HMBPP-pH = 4, bt-HMBPP-pH = 7 and bt-HMBPP-pH = 9). Each bar shows a different treatment which shows by the description written under each bar. Bars represented by *β*-estimation generated by the mixed model ± SE (*β*-lmer ± SE*)*; asterisks denote significant differences (*n* = 120, *p < 0.05, ** *p* < 0.01, ***p < 0.001).

We investigated the ability of *An. gambiae* s.l. females to feed on plant-based solutions of a beetroot juice with and without HMBPP as well as the effect of physiological pH on feeding activity. Low percentage of feeding was observed on the beetroot juice adjusted to neutral pH, while addition of 10 µM HMBPP to the feeding solution increased activity significantly (*χ*^2^_1_ = 12.05, *p* < 0.001) (Fig. 2a). The juice supplemented with HMBPP at pH = 7 produced the highest proportion of fed mosquitoes, whereas reducing the acidity to pH = 9 significantly decreased the feeding (*z* = 2.90 *p* = 0.01) (Fig. 2b). Acidification of the juice to pH = 4 obliterated the activity (Fig. 2b).

### Lipid/protein content maximizes mosquito feeding proportion with a plant-based solution

**Fig. 3:**
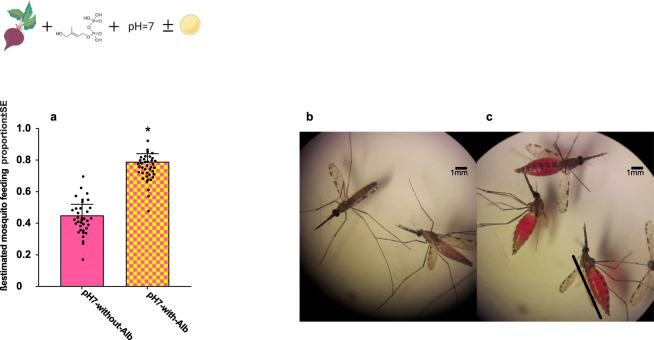
Mosquito feeding on a plant-based solution containing phagostimulant, HMBPP, adjusted to pH7, with and without lipid/protein addition. **a** Percentage of mosquitoes feeding within ten minutes on pH = 7 of plant-based solution with and without AlbuMAX^TM^ (pH7-with-Alb vs pH7-without-Alb) in *Anopheles* gambiae s.l. [*χ*^2^_1_ = 6.26, *p* = 0.01]. In this experiment, the left bar controls, and the right bar is the treatment. Bars represented by *β*-estimation generated by the mixed model ± SE (*β*-lmer ± SE); asterisks denote significant differences (*n* = 120, *P < 0.05, **P < 0.01, ***P < 0.001). **b**, **c** *An. gambiae s.l*. mosquitoes after feeding on plant-based mixtures varying in lipid content at physiological pH. Mosquitoes were exposed to feeding mixtures (**b**) without AlbuMAX^TM^ and (**c**) with AlbuMAX^TM^ for 10 min (scale bar: 1.0 mm).

We next optimised mosquito feeding proportion on a beetroot juice adjusted to pH = 7 and containing phagostimulant, HMBPP, by adding a lipid/protein source. Thereby, mosquitoes were exposed to feeding solutions with and without the lipid/protein source, (0.05%) AlbuMAX^TM^, for 10 min. Approximately 80% of *An. gambiae* s.l. females fed when provided AlbuMAX^TM^-supplemented artificial plant-based solution compared to 42% of those fed with meal without lipid/protein source (*χ*^2^_1_ = 6.28, *p* = 0.01) (Fig. 3a–c). When the solution containing AlbuMAX^TM^ without HMBPP was compared to the solution without AlbuMAX^TM^ and HMBPP, mosquito feeding proportion did not show any significant difference between these feeders (*χ*^2^_1_ = 6.86, *p* = 0.17). Notably, our data showed that activity of HMBPP as a phagostimulant is dependent on factors such as physiological pH of feeding solution and the serum lipid concentration.

### Supplementation of HMBPP to an optimised non-blood solution with synthetic toxin kills vectors

**Fig. 4:**
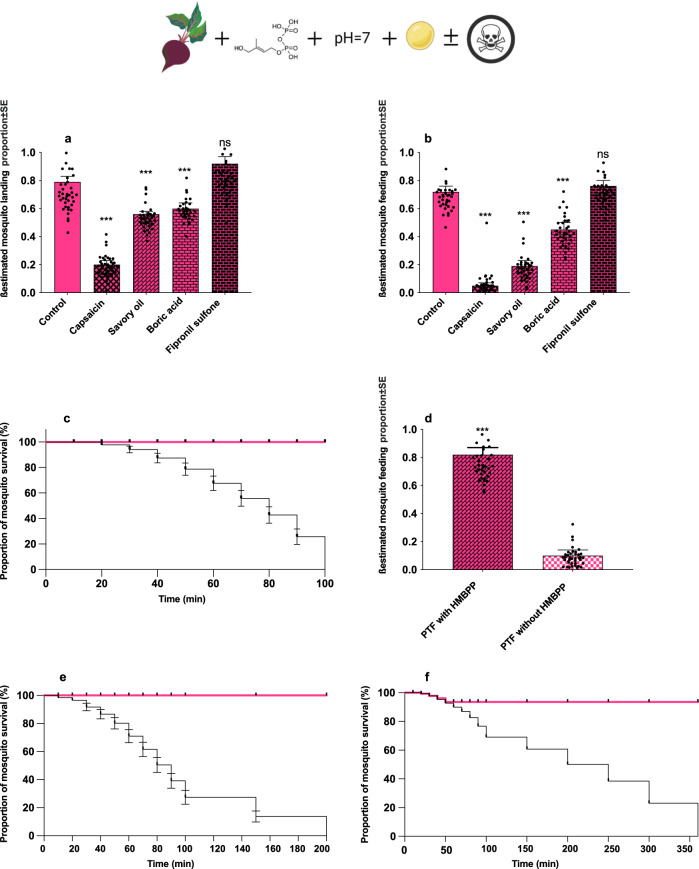
Supplementation of HMBPP in the optimised non-blood artificial solution with toxins tricks and kills vector. **a** Proportion of mosquito (*Anopheles gambiae* s.l.) landing on toxin-containing solutions compared to the control meal [capsaicin: *χ*^2^_1_ = 84.75, *p* < 0.001; Savory oil: *χ*^2^_1_ = 64.70, *p* < 0.001; boric acid: *χ*^2^_1_ = 90.35, *p* < 0.001; fipronil sulfone: *χ*^2^_1_ = 0.95, *p* = 0.29]. **b** Proportion of *An. gambiae* s.l. mosquito feeding on toxin treatments compared to the control meal. **c** Kaplan–Meier graph representing the survival of *An. gambiae* s.l.mosquitoes [%] subsequent to the feeding of fipronil sulfone (black line) and control (pink) during a 100 min time (time of total death in treatment cohort) frame. **d** Proportion of mosquitoes ingesting non-blood-feeding solution containing toxin fipronil sulfone (PTF) with and without the addition of HMBPP (*χ*^2^_1_ = 74.75, *p* < 0.001). **e** The survival of *Ae.aegypti* mosquitoes [%] subsequent to the feeding of fipronil sulfone (black line) and control (pink) during a 200 min time frame (time of total death in treatment cohort). **f** The survival of *Culex pipiens/Cx. torrentium* mosquitoes [%] subsequent to the feeding of fipronil sulfone (black line) and control (pink) during a 350 min time frame (time of total death in treatment cohort). Bars in panels (**a**), (**b**) and (**d**) are represented by *β*-estimation generated by the mixed model ± SE (*β*-lmer ± SE); asterisks denote significant differences (*n* = 120 in all experiments, *p < 0.05, **p < 0.01, ***p < 0.001; ns = non-significant).

Toxin feeding was carried out in order to find a potential compound that killed mosquitoes after ingestion. Therefore, the mosquitoes were offered one of four toxic formulations, which contain two natural and two synthetic toxins: namely capsaicin, savory oil, boric acid, and fipronil sulfone, in the optimised non-blood-feeding solution followed by an assessment of the landing, feeding and death rates of each treatment. A significantly lower proportion of mosquitoes landed on the feeders bearing capsaicin, savory oi, and boric acid compare to the proportion of mosquitoes landing on control while fipronil sulfone did not affect landings (capsaicin: *χ*^2^_1_ = 84.75, *p* < 0.001; savory oil: *χ*^2^_1_ = 64.70, *p* < 0.001; boric acid: *χ*^2^_1_ = 90.35, *p* < 0.001; fipronil sulfone: *χ*^2^_1_ = 0.95, *p* = 0.29). Even though mosquitoes landed on the membrane feeding containing capsaicin, few of them tasted it, most of them did not probe, and ingest this compound compare to control [capsaicin: *z* = 11.16, *p* < 0.001] (Fig. 4a, b). Lower proportions of mosquitoes feeding on solutions containing savory oil and boric acid were registered, compared to the proportion of mosquitoes feeding on the control solution [savory oil: *z* = −11.02, *p* < 0.001; boric acid *z* = 5.64, *p* < 0.001], while the difference in proportion of mosquitoes feeding on the solution containing fipronil sulfone was non-significant in comparison to those feeding on control [*z* = 0.75, *p* = 0.94] (Fig. 4a, b). No dead mosquitoes were observed in the 24 h following the feeding of mixtures supplemented with capsaicin, savory oil and boric acid, hence, the survival experiment was only conducted with optimised feeding mixture with or without fipronil sulfone. Based on the WHO insecticide susceptibility bioassay and previous studies^43^, mosquitoes are exposed to known concentrations of an insecticide for a fixed period of time, and the number of fatalities is recorded at least 24-h after exposure. Maximum 24-h monitoring of mosquito survival after exposing to various toxins is efficient for checking the range of the toxin knock-down and killing effects. In most mosquito pesticide evaluations, after 24 h 98–100% of mosquitoes exposed to effective toxins are dead^44^. The mosquito population was surveyed during the first 100 min subsequent to the feeding of fipronil sulfone (Fig. 4c, d). Compared to the control, the mosquito population was significantly reduced (*χ*^2^_1_ = 64.75, *p* < 0.001). The addition of HMBPP was key to the mosquito ingestion of the toxic solution (Supplementary Fig. 2), as in its absence, proportion of feeding drops significantly (*χ*^2^_1_ = 74.75, *p* < 0.001) from approximately 80 to 10% (Fig. 4d–f).

## Discussion

Vector-borne diseases are still a big burden for human and animal. Reducing transmission contributes to eliminating vector-borne diseases. There are two approaches to achieve this goal: one is to reduce the amount of pathogens and the other is to reduce the population of mosquito vectors. The combination of both approaches is clearly advantageous. The present study provides evidence that the chemical compound HMBPP (Supplementary Fig. 1) influences the feeding of several key vector mosquito species based on blood and non-blood-feeding mixtures. The selected mosquito vector species we used are associated with almost every major type of *Plasmodium* host-interaction systems from human to bird hosts. The variation among feeding proportion of mosquito species on blood without HMBPP (uninfected blood) can be defined by their habits, seasonality, abundance, and vectorial capacity. However, MEP pathway and its building blocks, including HMBPP is a conserved necessary pathway in all *Plasmodium* strains; *Plasmodium* survival depends on the operation of this pathway^45^. Supplementation of the blood with HMBPP reduced the vector feeding proportion variations between species, this phenomenon might help to determine how malaria is transmitted to and expressed in individual hosts and populations.

Supplementation of HMBPP in a plant-based artificial solution combined with a synthetic toxin entices and kills *Anopheles* vectors within ~100 min (Supplementary Fig. 5 and Fig. 4d–f). A recent study demonstrated an attraction as well as an increased phagostimulatory behaviour in *Anopheles gambiae* s.l. mosquitoes as a result of supplementation of HMBPP in human blood in the laboratory^41^. HMBPP has a great potential for increasing specificity of feeding based mosquito control techniques such as ATSB technique. ATSB, consisting of attractant, toxin, and sugar as a phagostimulant. Although ATSB is already implemented in some areas to suppress mosquito population^46^, ATSBs have a pronounced negative effect on non-target organisms including pollinators and other beneficial arthropods^23,24,47^. Therefore, the phagostimulant HMBPP might help to develop a selective toxic bait with an effective feeding/killing system for mosquitoes and greater selectivity.

To create a scalable mosquito toxic bait, blood needs to be replaced with a cheap and readily available liquid base, and beetroot juice serves as a good alternative for replacing human blood due to its dark pink colour. Fed mosquitoes have a pink abdomen which makes the assessment of feeding practicable in both the lab and field. In addition, it has been reported recently that beetroot peel can be deployed as an attractant for *Aedes aegypti* mosquitoes due to the beetroot-derived volatile compound, geosmin^48^. The proportion of feeding significantly improved when the pH of the feeding mixture was adjusted to neutral (pH = 7), whereas, notably fewer mosquitoes fed on the acidic and basic pHs feeding mixtures (Fig. 2b). The natural pH was preferred by the mosquitoes. *An. gambiae* s.l. natural food source is preferentially human blood which usually has a physiological pH level around 7.4, depending on the human’s diet^49^. The insect favours a diet close to natural physiological pH conditions. Furthermore, female mosquitoes ingest blood to support egg production and development^50,51^, hence we have investigated the effect of AlbuMAX^TM^ as a source of lipids, and proteins on feeding proportion of the beetroot juice-based mixture. That a higher proportion of mosquitoes fed on the mixture containing the lipid source AlbuMAX^TM^, compared to a non-supplemented meal revealed the need for factors such as protein and fatty acids for the phagostimulatory behaviour of the mosquitoes. Since albumin is an abundant protein present in serum^52^, AlbuMAX^TM^ is a good supplement due to its high content of bovine serum albumin (BSA) combined with lipids. It is also used as a replacement for serum in culturing of the malaria parasite *P. falciparum*^53^ in most laboratory settings. The feeding proportion is higher since the female mosquitoes need a protein/lipid source to nourish the eggs subsequent to mating^54^. Until to date, there is no deterrent effect for anthropophilic mosquitoes due to the presence of BSA^55^.

The experimental approach of feeding female *An. gambiae* s.l. mosquitoes with different compounds with potential toxicity in a feeding solution has challenges. Repellence and deterrence of a toxin are the major factors interfering with feeding process. Based on that, a less repellent and deterrent compound needs to be used in an attractive toxic bait (ATB) for achieving high attraction and feeding persistency of mosquitoes. Subsequent to landing on the host, the mosquito starts probing by penetrating the skin^56^ and therefore, the insect might be able to assess the presence of deterrent compounds, preventing or inhibiting the ingestion. A significantly lower proportion of mosquitoes landed on the feeders and probed non-blood solution supplemented with either capsaicin, savory oil or boric acid. This phenomenon could be due to repellent or antagonistic as well as ‘burning taste’ effects of toxins. Moreover, toxins could cause reactions with components in the feeding mixture forming repellents or deterrents. Several studies have reported that capsaicin acted as a repellent against insects^57–59^. However, this type of behaviour remains to be confirmed by applying different experimental designs that can prevent insects’ contact with capsaicin and rule out ‘burning taste’ effect which might cause the insect to move out of a treated area. In many animals including insects, capsaicin causes irritation, demonstrated as burning pain through chemoreceptors and nociceptors^57,60,61^ which affects foraging and food-averse migratory behaviour^58,59,62^. Nociceptors responding to heat and hazardous compounds have been identified in *Anopheles*, *Aedes* and *Culex* mosquito species^63,64^. Therefore, these evidences make it plausible that in our experiments the capsaicin acted as a deterrent against *An. gambiae* s.l. females when mosquitoes came in contact with the compound. At the beginning of the experiment, few females initiated probing making small holes in a feeder membrane but did not feed which we assume allowed subsequent females to detect capsaicin shortly after landing without probing and to fly away from the feeder. In contrast, no irritant but repellent and toxic effects have been reported towards *An. gambiae* mosquitoes exposed to savory oil at the highest 1% concentration tested^65^. In the present study, the 1:8 dilution of pure essential oil showed a significant reduction in landings compared to the control, suggesting a repelling or antagonistic effect. However, our experiment design was not able to distinguish between repellent effect^66,67^ of a toxin causing mosquitoes to move away from the source or the attraction-antagonistic effect^68^. Attraction antagonist targets odorant receptors, disrupting the odour-sensing function and decreasing mosquitoes’ ability to find a feeder but not repelling them. Notably, the antifeeding activity of the savory oil determined when the proportion of fed mosquitoes was three times lower compared to the proportion of landed females on the feeders bearing the non-blood artificial solution with savory oil. Currently, no antifeedant activity of savory oil produced from *Satureja montana* plants which is used in this study against insects has been reported, whereas essential oils isolated from related savory species *Satureja hortensis*^67^ L. and *Satureja pilosa* s.l. Velen^69^ showed a deterrent effect.

A significantly lower proportion of females landed and fed on the feeder providing the mixture supplemented with 1% of boric acid (dissolved in milli-q water) compared to the control. Boric acid as an inorganic poison is the most often used in toxic sugar baits against vector mosquitoes^23,70,71^, and no repellent, attraction-antagonistic and feeding-deterrent effects have been reported. Nonetheless, 1% of boric acid reported to reduce mosquito survival 55% within the subsequent 24 h monitoring period. The boric acid mechanism of action in attractive toxic sugar bait (ATSB) is assumed to be the disruption of the gut epithelium subsequent to ingestion by *Aedes aegypti* mosquitoes^72^. In some ATSB, 2% of boric acid was added producing a feeding mixture composed of chlorfenapyr 0.5% v/v, boric acid 2% w/v and tolfenpyrad 1% v/v, mixed in a guava juice-based bait^70^. In the laboratory, this mixture killed more than 90% of pyrethroid-susceptible *An. gambiae* s.s. and pyrethroid-resistant *An. arabiensis* and *Cx. quinquefasciatus*. However, in the field trial, mortality rates of the three ATSB treatments ranged from 41 to 48% against *An. arabiensis* and 36 to 43% against *Cx. quinquefasciatus*^70^. Another possible factor inducing tissue rupture in the gut subsequent to ingestion of a boric acid solution might be a highly increased osmotic pressure due to a higher concentration of sugar in the feeding solution as a trigger factor. For instance *Aedes aegypti* mosquitoes have aquaporins for balancing a certain range of osmotic pressure^73^. However, the concentration of the ingested free sodium ions might be too high in order to be balanced in a short time, causing a potentially not compensatable osmotic pressure and thereby leading to a possible tissue rupture. Based on that, the death of fed mosquitoes subsequent to the feed of boric acid in the beetroot mixture might not be induced due to the absence of an increased sugar amount, which may be the cause of an increased osmotic pressure and thus, tissue damage. Addition of fipronil sulfone to the feeding solution had no significant effect on landings and feeding of *An. gambiae* s.l. females compare to these types of behaviour registered to control. To date, there are no reports no effects on the feeding of mosquito^74,75^, and some other insects^76^ on fipronil-based baiting systems, although toxicity of the compounds was pronounced for *Aedes*, *Anopheles* and *Culex* mosquitoes^23^. Fipronil sulfone is already applied in ATBs targeting leaf-cutting ant colonies, fipronil causing a fast death in ants followed by the intake of the compound as well^77^. An evolutionary-conserved phagostimulant that enhances gorging can be combined with various mosquito control agents from different classes, biological (Bti), genetic (dsRNA) and chemo-toxic (boric acid), to enhance their efficacy for use in the control systems. Successful pairings will then be evaluated under field conditions, and prepared for integration into vector-control programmes, where appropriate.

Epidemiological aspects of malaria show the need for new vector-control approaches and efficient drugs to assure a reduction of the transmission, since parasite and vector resistance has already developed^3^. Parasite resistance against the artemisinin-based combination therapy has also been spreading vigorously in Southeast Asia^3,78,79^. To address the emerging resistance, the research for new drugs and vector-control strategies needs to be continued. HMBPP can underpin an advanced active toxin-feeding formulation for mosquitoes by enabling the use of a different feeding system. Moreover, HMBPP included in the beetroot juice feeding mixture could be combined with a toxin in an attractive toxin bait, supporting the development of new vector-control strategies. The toxin-feeding methods using beetroot mixture needs further testing and optimising. The beetroot mixture comprising fipronil sulfone taken up by *An. gambiae* mosquitoes and includes less sugar compared to other toxic baits approaches, thereby has the potential to serve as a more environmentally friendly toxic bait in future vector-control strategies. For field studies and strategies, a human odour blend can be used, increasing mosquito attraction towards the toxin-feeding station even further. More detailed work on new vector-control strategies is required in order to reduce vector-borne disease transmission, particularly for malaria on a large scale, and in natural settings.

## Methods

### Ethics

Human blood (type O) was provided in citrate-phosphate-dextrose-adenine anti-coagulant/preservative, and serum (type AB) was obtained from the Blood Transfusion Service at Karolinska Hospital, Solna, Sweden in accordance with the Declaration of Helsinki and approved by the Ethical Review Board in Stockholm (2011/850-32). The informed consent was obtained prior to blood draws at the hospital due to the blood transfusion regulation protocols.

### Materials

(*E*)-4-hydroxy-3-methyl-but-2-enyl pyrophosphate (HMBPP), capsaicin, savory oil, boric acid, fipronil sulfone and hydrochloric acid were purchased from Sigma-Aldrich (St. Louis, Missouri, USA); isopentenyl pyrophosphate (IPP), from Sigma-Aldrich (Tampa, USA, LC). The anti-coagulant/preservative, citrate-phosphate-dextrose-adenine was purchased from Vacuette (Greiner Bio-One Kremsmünster, Austria). 4-Aminobenzoic acid was purchased from Sigma-Aldrich Sweden (Stockholm, Sweden).

### Mosquito rearing conditions

Mosquitoes of the *Anopheles coluzzii*, *Anopheles arabiensis*, *Anopheles gambiae* s.s., *Aedes aegypti*, laboratory colonies were reared under insectary conditions and maintained in 27 ± 1 °C, 80 ± 1% humidity and a photoperiod of 12-h light:12-h dark cycle. Larvae were fed fish flakes (Goldfish Colour Sticks; Tetra, Germany). Emerged adults were fed ad libitum on 5% glucose solution, supplemented with 0.05% (w/v) 4-aminobenzoic acid, through soaked filters on top of the 2 ml tubes with soaked filter pads inside cages. Females of two morphologically similar, sympatric, sibling species *Culex pipiens* Linnaeus and *Culex torrentium* Martini were captured near Stockholm University campus and kept under laboratory conditions for 2–3 days with access to the glucose meal.

### Experimental set-up and feeding conditions

Ten minutes before experiments, the females were transferred into a cylinder-shape cardboard box (13-cm diameter, 10-cm height) top of which was covered with a net. Each beaker contained 20 mated, blood-starved but sugar-fed female mosquitoes around 5 to 7 days post emergence. Mosquito only had access to water in the 12 h before the initiation of the experiment. The feeding solution was filled into a feeder which was covered with a membrane (parafilm; Sigma-Aldrich, Germany) and heated to 36 ± 1 °C by a connection of the feeder with a warm water bath. The heat as well as the human volatiles transferred from a hand by rubbing feeder membrane on a skin were used as additional attractants for the mosquitoes. The hands of the person who carried out all feeding experiments have been washed with odourless soap just before the membranes were rubbed on the skin. The mosquitoes were fed for 10 min followed by an assessment of the landing and feeding rate. The landing is assessed using the number of mosquitoes that landed on the feeder, and started the investigation on the feeder. Feeding rate (proportion) was recorded based on the number of mosquitoes had full red gut at each time point of the experiment.

### Blood-feeding experiments

Ten to twenty mosquitoes of the same species (*An. coluzzii: n* = 20, *An. arabiensis: n* = 20, *An. gambiae* s.s.: *n* = 20, *Ae. aegypti: n* = 20 and *Cx. pipiens/Cx. torrentium*: *n* = 10) were placed in each cage covered with netting, and fed either on 1 ml of control RBCs or HMBPP-RBCs. Each group was allowed to feed on its own separated membrane feeder for 10 min, the average time for each *Anopheles* mosquito engorgement is 10 min^80^. For each group, the number of fully fed mosquitoes was recorded every minute. RBCs were washed with Roswell Park Memorial Institute (RPMI) medium and stored in RPMI at 50% haematocrit at 4 °C. In each feeding trial, RBCs stored in RPMI were centrifuged at 2500 × *g* for 5 min followed by replacement of the medium with AB serum for a final haematocrit of 40%. HMBPP (stock concentration at 4 mM in Nanopure water, stored at −80 °C) was diluted to 10 μM in 1 ml RBC suspension, and the corresponding volume of Nanopure water was added to the control RBCs. All experiments were, unless otherwise stated, conducted on 5–7 days post-emergence female mosquitoes maintained in separated cages and fed RBCs either with or without HMBPP for 10 min. All experiments were performed in triplicate.

### Feeding on plant-based solution

The feeding mixture comprised filtrated (0.22 µm) beetroot juice diluted (1:2) with an AlbuMAX^TM^ II solution (5 mg/ml, dissolved in milli-q water). The pH of the solution was adjusted to 7 by adding required amount of hydrochloric acid followed by a supplementation of the phagostimulant HMBPP (10 µM). In feeding experiments, artificial solution (beetroot, pH = 4, 7 and 9), either with or without HMBPP, were offered to mosquitoes during a 10 min feeding window, and the number of fed mosquitoes recorded. For the proportion of mosquito feeding (%), the number of fed mosquitoes (engorged red abdomen) were set in relation to the total number of mosquitoes in the box.

### Feeding mixture and toxin contents

The optimised feeding mixture contained filtrated beetroot juice diluted 1:2 in an AlbuMAX^TM^ II solution (5 mg/ml) and complemented with the phagostimulant HMBPP (10 µM). The pH was adjusted to 7. Subsequently, toxin was added to the mixture, respectively. Based on the previous literatures, following concentrations of the toxins were used: 0.5% of capsaicin (dissolved in RPMI and 70% ethanol), 100 µl of 1:8 diluted savory oil solution, from *Satureja montana* (dissolved in RPMI and 70% ethanol), 1% of boric acid (dissolved in milli-q water) and 100 µM of fipronil sulfone (dissolved in propylene glycol).

### Toxin-feeding procedure and determination of survival proportion

During the toxin-feeding experiment, the blood-starved, only sugar-fed mosquitoes were fed 1.5 ml of the feeding mixture comprising the respective toxin (mixture heated to 36 ± 1 °C) for 10 min. Subsequent to the feeding, a 5% glucose solution was provided, and the insects were monitored every 30 min for the first 6 h, followed by a 24 h check. Landing, feeding and survival proportion of mosquitoes were determined. The landing proportion was assessed by the fraction of mosquitoes that landed and remained on a feeder within the first 10 min in the cup. The number of fed mosquitoes (red abdomen) in relation to the total number of mosquitoes was used to determine the feeding proportion. The number of dead mosquitoes per total number of mosquitoes was used for assessing the survival proportion. The concentration HMBPP in all experiments were 10 μM.

### Statistics and reproducibility

General Linear Mixed Model (GLMM) statistical modelling was used to corroborate the validity of results based on the whole data set by including the effect of replications (experimental blocks), including weighting for multiple replications. In all analyses, the effect of the main experimental effects (e.g. treatment [HMBPP-RBCs vs RBCs]^81^) was investigated while controlling for variation in experimental replication (random variable). For all results, the significance of all explanatory effects was evaluated by using likelihood ratio test (LRT). Analyses were performed using R statistical software (R Core team^82^: R v.3.2.3 and RStudio 1.1.463^37^). Generalised Linear Mixed Models (GLMM, R statistical software v. 3.2.3) assuming a binomial distribution were used to test the effect of treatment and expermetal replication (random variable) on the response variable (accumulative feeding proportion), between different species variation (5 main vectors) assays (Cox proportional hazards model [Cox-PHZ]; survminer package, R, v. 3.2.3). Time-series analyses were used for estimating the accumulative proportion of mosquito feeding. In these experiments, we had one fixed explanatory variable under name of treatment (HMBPP-RBCs vs RBCs) as well as the effect of time. For calculating the cumulative probability of feeding (accumulative feeding proportion [%]), [Cox-PHZ] was used, which generally presented by Kaplan–Meier curves as it was showed in Fig. 1. In other words, this model allowed us to examine how specified factors (fixed factor treatment [HMBPP vs Control RBCs] and random variable [experimental treatment]) influence the accumulated probability of a particular event happening (e.g., feeding) at a particular point in time (each minute until 10 min in total).

In this study, we included at least two variables: 1-Treatment, i.e. the HMBPP (main effect) and 2-Experimental blocks, i.e. the experimental replicates (random effect). In all analyses, Treatment (HMBPP or (2*E*)-2-methylbut-2-ene-1,4-diol) was investigated as the main effect of interest. All data conformed to the assumptions of the test (normality and error homogeneity). The landing, proportion of feeding on (2*E*)-2-methylbut-2-ene-1,4-diol, as well as optimising plant-based feeding solution, and survival proportion of mosquitoes exposed to control and toxin treated feeding mixtures were also analysed by linear mixed-effected model (lmer) via maximum likelihood teas using the statistical programme R (version 3.6.1) in the RStudio platform. Therefore, the rates were calculated as described prior to analysing the results with GLMM and the lmer function as described for Figs. 2–4. The *β*-estimated values were estimated in all final significant models and presented in the graphs (GLMM: lmer function). The duration of survival over time after feeding on toxic solution [time-to-death] was analysed using the Cox-PHZ; with time of follow-up feeding as the main covariate for evaluating the proportion of survival as the outcome, with experimental replication incorporated as a random effect [frailty function]. In all mixed models, a maximal model was built that included fixed effects plus the random effects of the experimental replicates. All graphical representation and statistical analyses were performed using Graphpad Prizm version 6.0c software or R (v.3.2.3 and Rstudio Version 1.1.463 – © 2009–2018). *P*-values of *p < 0.05, **p < 0.01, ***p < 0.001 and ****p < 0.0001 were deemed to be statistically significant.

### Reporting summary

Further information on research design is available in the Nature Research Reporting Summary linked to this article.

## Supplementary information

Supplementary Information

Supplementary Data 1

Reporting Summary

## Data Availability

All data supporting the findings of this study are available within the article and its Supplementary Information file, Supplementary Data 1, and are available from the corresponding author upon request.
