# Supplementary material for: Plasmodium metabolite HMBPP stimulates feeding of main mosquito vectors on blood and artificial toxic sources
Source: Commun Biol. 2021 Oct 7;4:1161. doi: 10.1038/s42003-021-02689-8 (PMC8497504; doi:10.1038/s42003-021-02689-8)

Supplementary Information for

Plasmodium metabolite HMBPP stimulates feeding of main mosquito vectors on blood and artificial toxic sources

Viktoria E. Stromsky[1]†, Melika Hajkazemian[1]†, Elizabeth Vaisbourd[1], Raimondas Mozūraitis[2,3] & S. Noushin Emami[1,4,5]*

[1]Department of Molecular Biosciences, Wenner-Gren Institute, Stockholm University, Stockholm, Sweden.

[2]Laboratory of Chemical and Behavioural Ecology, Institute of Ecology, Nature Research Centre, Vilnius, Lithuania.

[3]Department of Zoology, Stockholm University, Stockholm, Sweden.

[4]Molecular Attraction AB, Elektravägen 10, 126 30 Hägersten, Stockholm, Sweden.

[5]Natural Resources Institute, FES, University of Greenwich, London, UK.

† These authors contributed equally to this work

*Corresponding author. E-mail: noushin.emami@su.se

This PDF file includes:

Supplementary Figures 1 - 5

Supplementary Figure 1. The structure of HMBPP and ATP phagostimulants

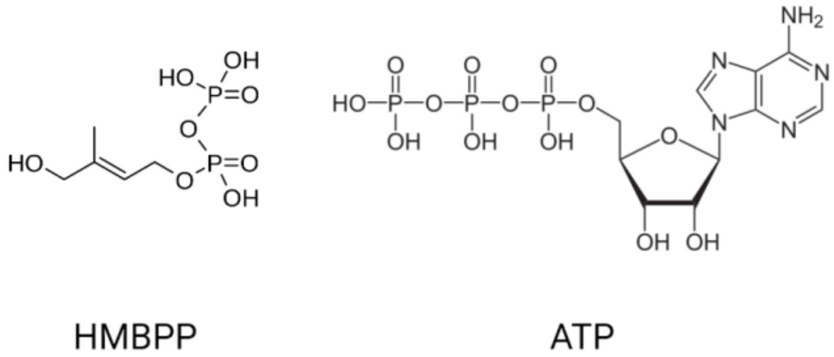

Supplementary Figure 2. After optimising the feeding/killing solution, *An. gambiae* s.l. mosquitoes also killed by toxic-feeding on plant-based mixture at physiological pH with addition of 1% blue food colour. Mosquitoes were exposed to feeding mixtures for 10 min (scale bar: 1.0 mm).

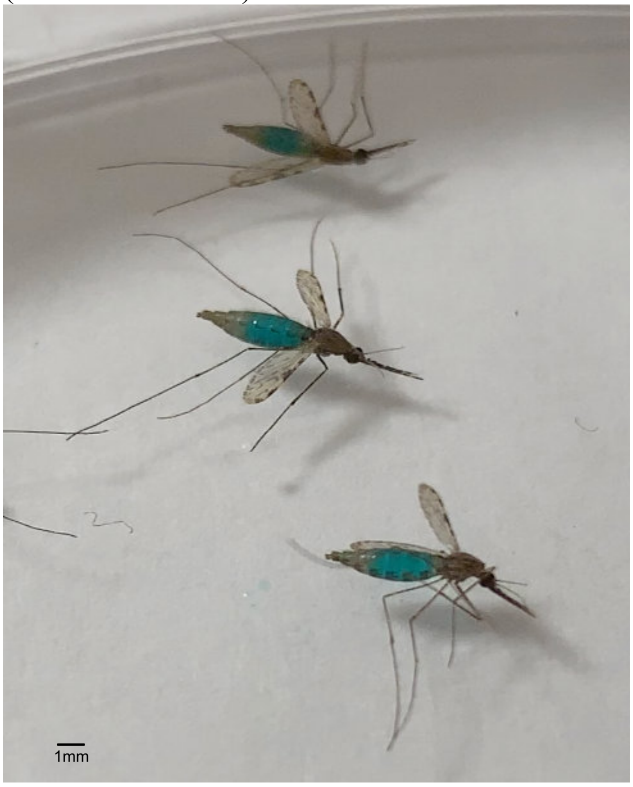

Supplementary Figure 3. Direct effect of Diol on *An. gambiae* s.l. feeding proportion (%), [β-lmer ± SE= 0.42 ± 0.05].

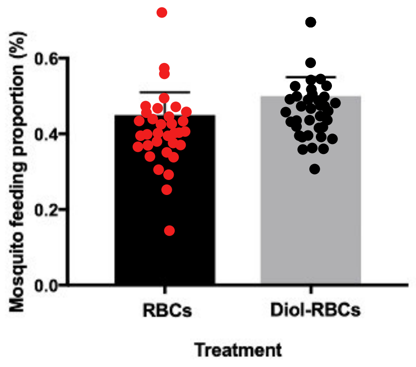

Supplementary Figure 4. Accumulative landing and probing proportions (initiating of feeding %) in fine main mosquito vectors. Mosquitoes exposed to human blood (control: RBCs + serum) for 60 seconds. Each level of estimation generated from cox proportional hazard model ± SE.

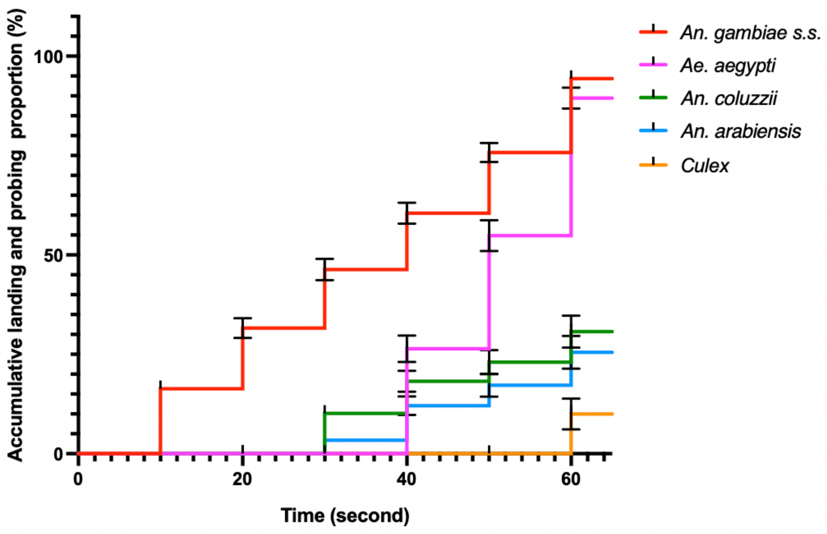

Supplementary Figure 5. Graphical abstract of the study

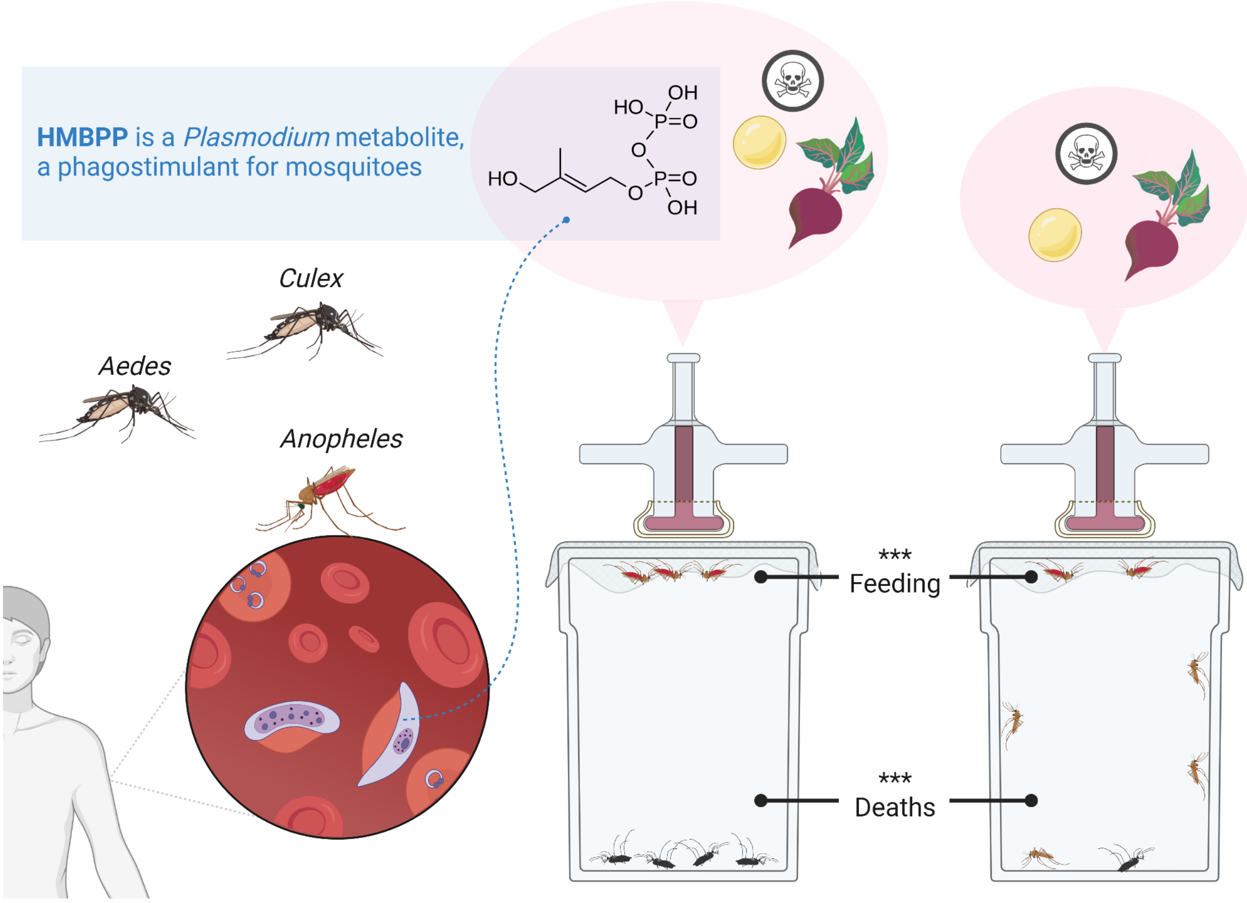

Supplement: Supplementary file 1 — Supplementary Information [file 42003_2021_2689_MOESM1_ESM.pdf]
