# Supplementary material for: Plasmodium metabolite HMBPP stimulates feeding of main mosquito vectors on blood and artificial toxic sources
Source: Commun Biol. 2021 Oct 7;4:1161. doi: 10.1038/s42003-021-02689-8 (PMC8497504; doi:10.1038/s42003-021-02689-8)
Supplement: Supplementary file 3 — Reporting Summary [file 42003_2021_2689_MOESM3_ESM.pdf]

nature research

Corresponding author(s): Dr. S. Noushin Emami

Last updated by author(s): Sep 3, 2021

Reporting Summary

Nature Research wishes to improve the reproducibility of the work that we publish. This form provides structure for consistency and transparency in reporting. For further information on Nature Research policies, see our Editorial Policies and the Editorial Policy Checklist.

Statistics

For all statistical analyses, confirm that the following items are present in the figure legend, table legend, main text, or Methods section.

n/a	Confirmed	
☐	☒	The exact sample size (n) for each experimental group/condition, given as a discrete number and unit of measurement
☐	☒	A statement on whether measurements were taken from distinct samples or whether the same sample was measured repeatedly
☐	☒	The statistical test(s) used AND whether they are one- or two-sided<br>*Only common tests should be described solely by name; describe more complex techniques in the Methods section.*
☐	☒	A description of all covariates tested
☐	☒	A description of any assumptions or corrections, such as tests of normality and adjustment for multiple comparisons
☐	☒	A full description of the statistical parameters including central tendency (e.g. means) or other basic estimates (e.g. regression coefficient) AND variation (e.g. standard deviation) or associated estimates of uncertainty (e.g. confidence intervals)
☐	☒	For null hypothesis testing, the test statistic (e.g. F, t, r) with confidence intervals, effect sizes, degrees of freedom and P value noted<br>*Give P values as exact values whenever suitable.*
☒	☐	For Bayesian analysis, information on the choice of priors and Markov chain Monte Carlo settings
☒	☐	For hierarchical and complex designs, identification of the appropriate level for tests and full reporting of outcomes
☒	☐	Estimates of effect sizes (e.g. Cohen's d, Pearson's r), indicating how they were calculated

Our web collection on statistics for biologists contains articles on many of the points above.

Software and code

Policy information about availability of computer code

Data collection	no software was used for the data collection
Data analysis	All statistical analyses have been done using Generalized Linear Mixed Models (GLMM), and analyses were performed in R statistical software(V.3.2.3 and Rstudio v. 1.1.463). Visualisation were performed using GraphPad Prism (Licence 2020).

For manuscripts utilizing custom algorithms or software that are central to the research but not yet described in published literature, software must be made available to editors and reviewers. We strongly encourage code deposition in a community repository (e.g. GitHub). See the Nature Research guidelines for submitting code & software for further information.

Data

Policy information about availability of data

All manuscripts must include a data availability statement. This statement should provide the following information, where applicable:

- Accession codes, unique identifiers, or web links for publicly available datasets
- A list of figures that have associated raw data
- A description of any restrictions on data availability

Identifiers and the list of figures are included in the manuscript and supplementary documents

Field-specific reporting

Please select the one below that is the best fit for your research. If you are not sure, read the appropriate sections before making your selection.

☒ Life sciences ☐ Behavioural & social sciences ☐ Ecological, evolutionary & environmental sciences

For a reference copy of the document with all sections, see nature.com/documents/nr-reporting-summary-flat.pdf

Life sciences study design

All studies must disclose on these points even when the disclosure is negative.

Sample size	We used 120 mosquitoes per each experiment (20 mosquitoes per pot * 6 replications) for the laboratory studies. Sample size calculation was performed based on estimated parameters (such as variance and marginal error) and 95% confidence interval which were obtained from our previous studies.
Data exclusions	There are no data exclusion in this study
Replication	Each experiment has been replicated 4-6 times due to verification of the reproducibility of the findings (more information in the method section). All attempts at the replication were successful with the statistical models (mixed effect models) showed highly significant out puts.
Randomization	All samples (mosquitoes) have been collected in completely randomized method from the bigger colony populations. In two choice feeding assay, the environmental effects have been also controlled by rotation of the treatments in each replicate.
Blinding	All studies were performed using blinded strategy. For example, the percentage of feeding success calculated by the scientists without revealing whether the feeder contained the treatment or it was holding the control solution.

Reporting for specific materials, systems and methods

We require information from authors about some types of materials, experimental systems and methods used in many studies. Here, indicate whether each material, system or method listed is relevant to your study. If you are not sure if a list item applies to your research, read the appropriate section before selecting a response.

Materials & experimental systems

n/a	Involved in the study	
☒	☐	Antibodies
☒	☐	Eukaryotic cell lines
☒	☐	Palaeontology and archaeology
☐	☒	Animals and other organisms
☒	☐	Human research participants
☒	☐	Clinical data
☒	☐	Dual use research of concern

Methods

n/a	Involved in the study	
☒	☐	ChIP-seq
☒	☐	Flow cytometry
☒	☐	MRI-based neuroimaging

Animals and other organisms

Policy information about studies involving animals; ARRIVE guidelines recommended for reporting animal research

Laboratory animals	Mosquito colonies in MBW, Stockholm University (Sweden)
Wild animals	N/A
Field-collected samples	N/A
Ethics oversight	Colonised mosquitoes have not been considered under any Ethical oversight yet.

Note that full information on the approval of the study protocol must also be provided in the manuscript.
